# Biopolymer Green Lubricant for Sustainable Manufacturing

**DOI:** 10.3390/ma9050338

**Published:** 2016-05-05

**Authors:** Shih-Chen Shi, Fu-I Lu

**Affiliations:** 1 Department of Mechanical Engineering, National Cheng Kung University (NCKU), No. 1 University Road, Tainan 70101, Taiwan; 2 Institute of Biotechnology, College of Bioscience and Biotechnology, National Cheng Kung University, Tainan 701, Taiwan; fuilu@mail.ncku.edu.tw; 3 Department of Biotechnology and Bioindustry Sciences, College of Bioscience and Biotechnology, National Cheng Kung University, Tainan 701, Taiwan

**Keywords:** biopolymer, green lubricant, HPMC, sustainable manufacturing, transfer layer, biocompatibility, toxicity, zebrafish embryo

## Abstract

We report on the preparation of a biopolymer thin film by hydroxypropyl methylcellulose (HPMC), which can be used as a dry green lubricant in sustainable manufacturing. The thin films were characterized through scanning electron microscopy, energy-dispersive spectroscopy, and Raman spectroscopy; the films showed desirable levels of thickness, controllability, and uniformity. Tribology tests also showed desirable tribological and antiwear behaviors, caused by the formation of transfer layers. Zebrafish embryo toxicity studies showed that HPMC has excellent solubility and biocompatibility, which may show outstanding potential for applications as a green lubricant. The results of the present study show that these techniques for biopolymer HPMC provide an ecologically responsible and convenient method for preparing functional thin films, which is particularly applicable to sustainable manufacturing.

## 1. Introduction

The United Nations Framework Convention on Climate Change signed during the 2015 United Nations Climate Change Conference in Paris has made carbon reduction and energy conservation an issue to be addressed by all countries around the globe. To facilitate the most efficient use of resources, product designs and manufacturing philosophies that focus on “reducing” the use of resources, “reusing” resources, and “recycling” resources (*i.e.*, the 3Rs) are now widely discussed. To meet 3R demands, manufacturers proposed concepts of sustainable manufacturing and green lubricants, in which the idea of 3R is incorporated into the research and development of processing-related technologies. Major development directions of such technologies include green lubricant materials [1,2,3], dry coating methods such as diamond-like carbon films [4,5,6,7], and minimum quantity lubrication [8,9,10].

Green lubricant technologies [11,12] are a type of lubrication technology that reduces abrasion and wear, saves energy, is recyclable, and does not cause harm to the environment. To prevent the production of lubricants from having a negative effect on the environment and creating biologically toxic substances, efforts have been made to research and develop environmentally friendly lubricants [13,14] such as phosphorous and/or heavy metal-free materials. Researchers began studying lubricating oils that were non-petrochemical-based and derived from nature; such oils featured natural and outstanding bioavailability and biodegradability, making them an excellent choice as green lubricants. Examples of such oils include natural vegetable oil [15], rice bran oil [16], rapeseed oil [17], and coconut oil [2]. Natural oils display favorable scalability; research has shown that by adding appropriate additive(s), natural lubricating oils can demonstrate lubrication properties identical to those of petrochemical lubricants [18]. In addition, natural oils possess excellent lubrication effects and contain non-toxic and pollution-free lubricating substances [19,20,21,22]. Lubrication tests had also been performed in which original tissue fluids were replaced with solutions such as biopolymers (e.g., hyaluronic acid) [23,24] and mucins found in different animal body parts [25]. In addition, by mixing thickeners (e.g., guar gum, antler gum, and locust bean gum; most of which are biopolymers) with water, hydrocolloids can be obtained. The lubrication statuses of these hydrocolloids have been frequently discussed and simulated [25,26,27,28,29]. Concerning hydroxypropyl methylcellulose (HPMC), it is a biodegradable material that is environmentally and biologically friendly [30]. It does not contain sulfides and heavy metals, displays favorable physical stability under normal conditions, and is chemically inert to most additives. Therefore, it is used in human antibacterial [31] and drug delivery-related studies [32]. Because HPMC can be easily formed into films, it was made into dry films with a lubricating property [33]. This enabled us to solve the problem encountered by most lubricants in which lubricant viscosity changed over time, hindering lubrication effectiveness. The aim of the present investigation was to determine the tribological behavior of biopolymer HPMC thin film as a dry lubricating layer. The favorable characteristics of the HPMC make it an exceptionable candidate material for coatings on cutting tools as a future green lubricant.

To confirm that the biopolymer used in the present study has minimal environmental toxicity, we utilized zebrafish embryos. The zebrafish is a common animal model for toxicity assays [34]. According to the protocol for acute toxicity testing with the zebrafish embryo, we analyzed the potential toxicity of HPMC [11,35].

## 2. Results and Discussion

### 2.1. Film Characterization

**Figure 1:**
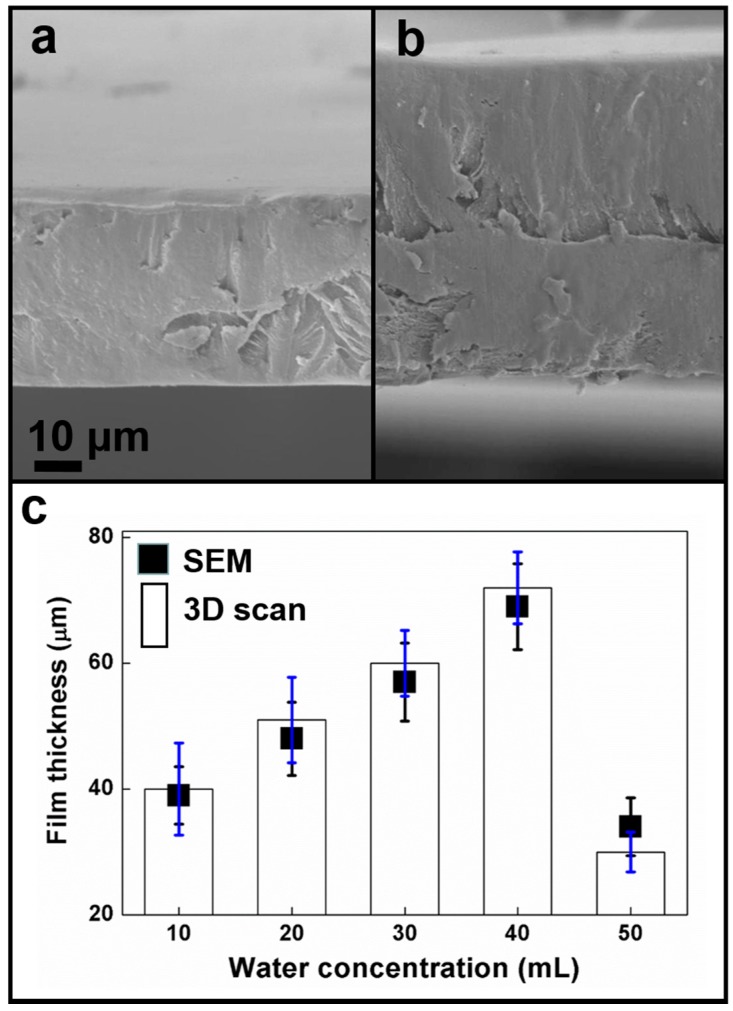
Cross-sectional SEM images of thin film at (**a**) 40 μm; and (**b**) 70 μm thickness; (**c**) the thickness variation diagram measured by SEM and 3D scanner.

According to the cross-sectional SEM images, the cross-sectional surface and top surface of HPMC film was smooth and uniform as shown in Figure 1. Film thickness was adjusted (40 μm–70 μm) by adjusting the water content. According to the material datasheet, when the water to alcohol ratio ranged from 20%–100%, HPMC powder dissolved completely and the solution was transparent. Optimal solubility was reached when the HPMC content to solvent ratio was 1:28, signifying that for 5 g of HPMC, the optimal solubility would be achieved by mixing it with 40 mL of water and 100 mL of alcohol. At this ratio, the mixture would produce the thickest film; both increases in solvent content and decreases in solute content would result in reduced film thickness. However, according to a previous study [36], the lubrication effect of HPMC is not influenced by film thickness. Only the lubrication duration is affected: the thicker the film is, the longer the lubrication duration becomes.

### 2.2. Raman Spectroscopy and EDS Analysis

**Figure 2:**
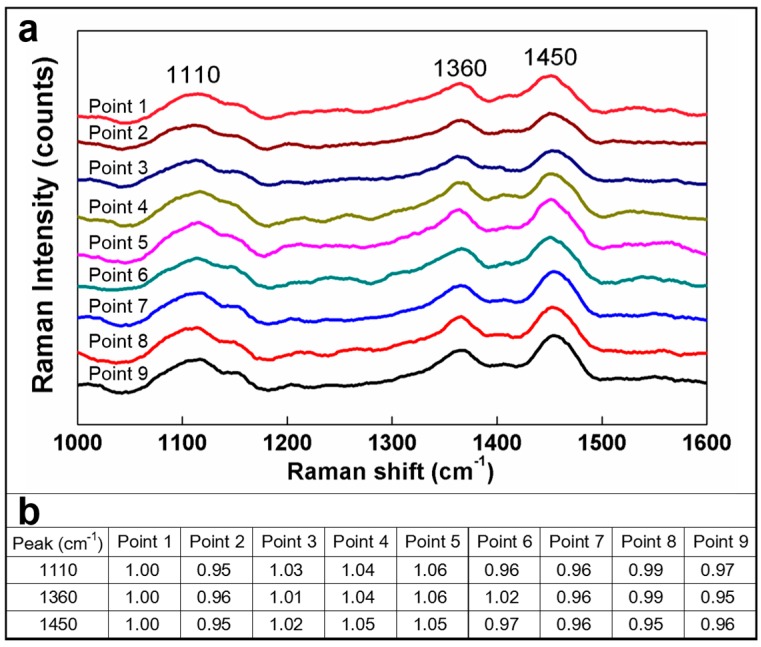
Analyzing film uniformity using the Raman spectrum. (**a**) The peak value intensities; (**b**) The detailed ratios.

To fully understand the film uniformity of the HPMC, this study performed a Raman spectrum analysis in addition to the cross-sectional images of illumination obtained using the SEM. The Raman spectrum intensity distribution analysis was employed to assess film uniformity. Three major characteristic peaks, namely, 1110 cm*^−^*^1^, 1360 cm*^−^*^1^, and 1450 cm*^−^*^1^ [37], were assigned for symmetric C-O-C [38], COH bending [39], and CH_2_ scissor [40] functional groups, respectively, as shown in Figure 2.

The three characteristic peak value intensities are compiled in Figure 2b. To facilitate comparisons between the characteristic peak value intensities, we set the peak value intensity at Point 1 as “1” and compared the remaining eight peak value intensities in relation to Point 1. The ratios are presented in Figure 2b. According to the data, all three characteristic peaks showed highly similar characteristic peak locations. Moreover, the intensity differences among the three characteristic peaks fell within 5%. Such a result confirmed that the HPMC demonstrated favorable uniformity. Both Figure 1 and Figure 2 show smooth and uniform films. Next, the surface activity of the HPMC during lubrication was explored. A previous study [36] found flat residues on the surface of silicon substrates after lubricants had been worn through. Therefore, in this study, SEM and EDS experiments were performed to find out the surface status of HPMC during lubrication.

**Figure 3:**
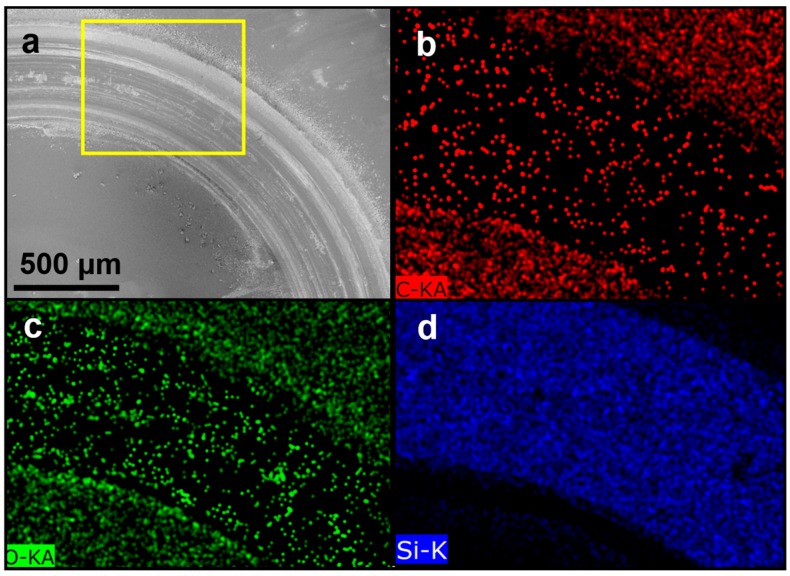
(**a**) SEM image of the wear mark on the HPMC on silicon substrate; EDS element mapping for (**b**) C; (**c**) O; and (**d**) Si signals in yellow marked area.

Figure 3a shows a low-magnification scanning electron microscope (SEM) image of the wear mark. The yellow box in Figure 3a was enlarged to facilitate an energy dispersive spectroscopy (EDS) element mapping, as shown in Figure 3b–d. Figure 3a shows a circular wear mark on the test specimen that underwent a lubrication test, and that the wear mark contained some material residues. The yellow box in Figure 3a was enlarged to facilitate an EDS analysis. Figure 3b–d respectively show the results of EDS element mapping for carbon (C), oxygen (O), and Silicon (Si) signals. Figure 3b,c show that the wear marks contain few C and O signals from the HPMC, indicating that during lubrication, the HPMC was removed layer by layer over time. Favorable lubrication was achieved through gradual removal of the HPMC material. HPMC residues remained on the silicon substrate before the HPMC was completely removed.

**Figure 4:**
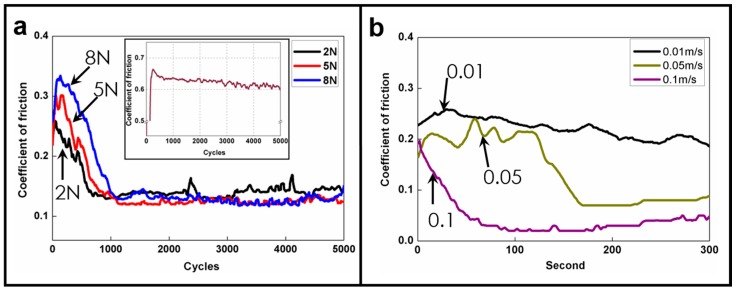
(**a**) Lubrication test result using varying loads under 0.01 m/s rotation speed; (**b**) lubrication test results using varying speeds. Inset shows the COF of bare Si as reference.

According to Figure 3d, the HPMC had been worn through (*i.e.*, removed from the surface of the silicon substrate) over a long period of use, resulting in the silicon substrate showing significant Si signals during EDS element mapping. Moreover, Figure 4 shows that the coefficients of friction (COFs) of the silicon substrate remain low, which is because the C and O signals remain in the wear mark (as shown in Figure 3c,d). This indicated that residues of HPMC on the silicon kept protecting the substrate, reduced the wear of silicon, and maintained low COFs.

**Figure 5:**
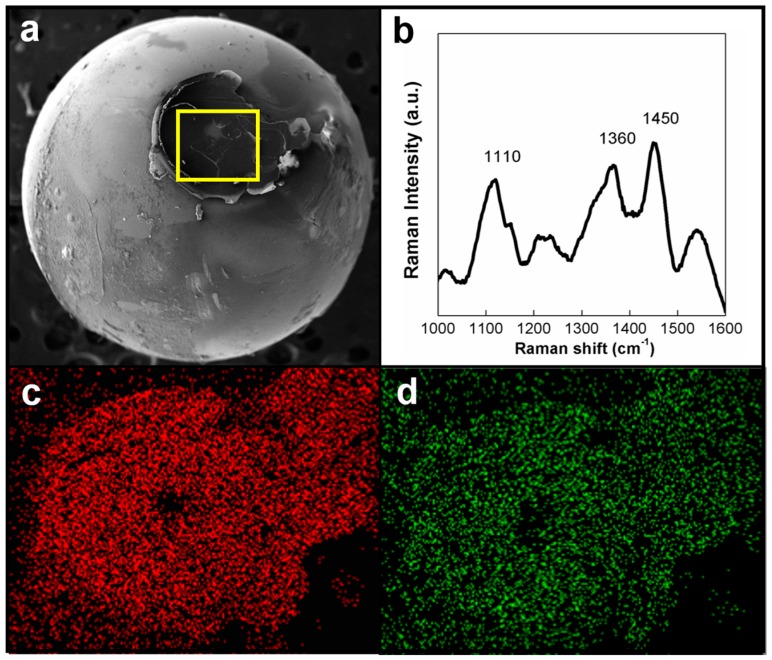
(**a**) SEM surface image of a chrome steel ball; (**b**) Raman spectrum analysis of a chrome steel ball; (**c**) EDS element mapping for C signals on a chrome steel ball; and (**d**) EDS element mapping for O signals on a chrome steel ball.

To find out where the HPMC had been removed to and the reason why the silicon substrate was able to maintain low COFs at the later phase of lubrication, this study analyzed the chrome steel ball used to scrape the silicon substrate. Figure 5a shows an SEM image of the surface characteristics of said chrome steel ball, in which a layer of lubricant was clearly observed on the surface. The yellow box in Figure 5a was subsequently analyzed using the Raman spectrum analysis and the results are presented in Figure 5b, which show the HPMC characteristic peaks [37].

The results indicated that during lubrication, the HPMC was transferred from the surface of the silicon substrate to that of the chrome steel ball, forming a protective layer between the two objects and leading to an HPMC–HPMC lubricating behavior. Considering that most polymers have a self-lubricating effect [41], it is the reason that the HPMC film was able to maintain low COFs and enhance its wear resistance. Figure 5c,d respectively present result of EDS element mapping of the C and O signals on the surface of the chrome steel ball. The two figures show a result identical to that observed in Figure 5b, that is, the C and O signals were the residues of the HPMC film. This finding verified that the HPMC was indeed transferred from the surface of the silicon substrate to that of the chrome steel ball as a result of the pressure experienced by the substrate film coating during the lubrication process, thus causing low and stable COFs and favorable wear resistance [42,43,44].

### 2.3. Tribology Test

A pin-on-disk tribotest was adopted to analyze the coatings synthesized using HPMC. To facilitate the analysis, the first and last 50 data cycles were averaged, as displayed in Figure 4. This figure illustrates the influences of loading and rotation speed on the tribological properties of the coatings. Figure 4a shows that the COFs were marked high during the initial stage of the wear test. For all three loading types, the COFs achieved a friction coefficient of 0.14 after stabilization. This result suggests that HPMC possesses superior lubrication characteristics compared with the friction coefficient of 0.6 (inset of Figure 4a) produced by Si under dry grinding. The COFs declined drastically to 0.14 and steadied when the number of cycles reached 1000 for all three loading types. The reason was the formation speed of an HPMC transfer layer [45,46]. Under identical speeds, the transfer speed of a transfer layer is similar. Therefore, stable, low friction values were obtained around the same time for all three load types. In addition, higher loadings during the initial stage created higher COFs because of higher contact stress, COFs were no longer influenced by contact stress once a transfer layer had been formed.

Figure 4b shows the lubrication test results using varying speeds, in which the results display trends similar to those in Figure 4a: the COFs were marked high during the initial stage of the wear test because of the lack of a transfer layer, but the COFs declined once a transfer layer had been formed. Furthermore, when the sliding speed increased, the COFs declined at an even faster rate because an increased rotational speed resulted in the quicker formation of the transfer layer, thus achieving a lubrication effect. Increased rotational speed also signified increased material contact frequency and quicker and thicker transfer layer formation [41,43], resulting in quicker achievement of favorable and stable lubrication properties.

### 2.4. Zebrafish Embryo Toxicity Test

**Figure 6:**
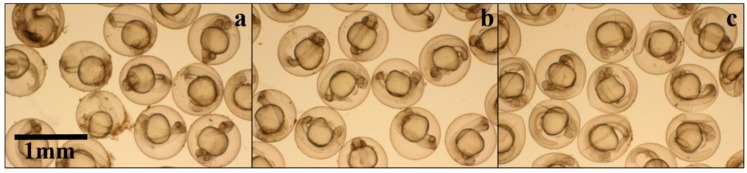
Phenotypes of zebrafish embryos at 24 hpf after treatment with different concentrations of (**a**) 0%; (**b**) 0.5%; (**c**) 1% HPMC.

At 24 h post fertilization (hpf) after incubation in HPMC according to the guidelines of the Fish Embryo Acute Toxicity (FET) test, treated embryos did not show any phenotype differences between groups, including coagulation of the embryos, lack of somite formation, or non-detachment of the tail (Figure 6). However, the chorions displayed some shrinkage in the embryos that had been incubated in 1% HPMC. This may have been caused by the differences of osmotic pressures inside and outside the chorions (hyperosmotic). Notably, rates of spontaneous body contraction were higher in the 1% HPMC incubated embryos (5.8 compared to 1.2 times/min in non-treated embryos, *n* = 5 for each treatment). This may have been caused by the shrinkage of the chorion; if the chorion shrank and then touched the surface of the embryo, then that may have caused reflection contractions [47].

**Figure 7:**
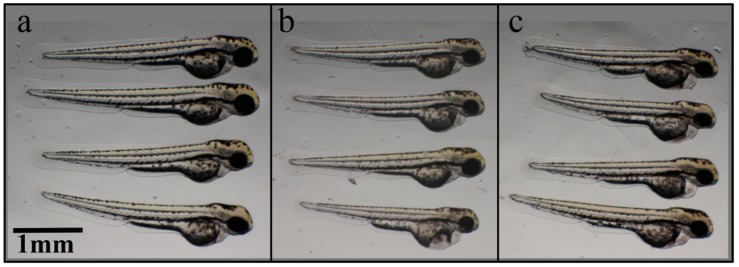
Phenotypes of zebrafish embryos at 48 hpf after treatment with different concentrations of (**a**) 0%; (**b**) 0.5%; (**c**) 1% HPMC.

**Figure 8:**
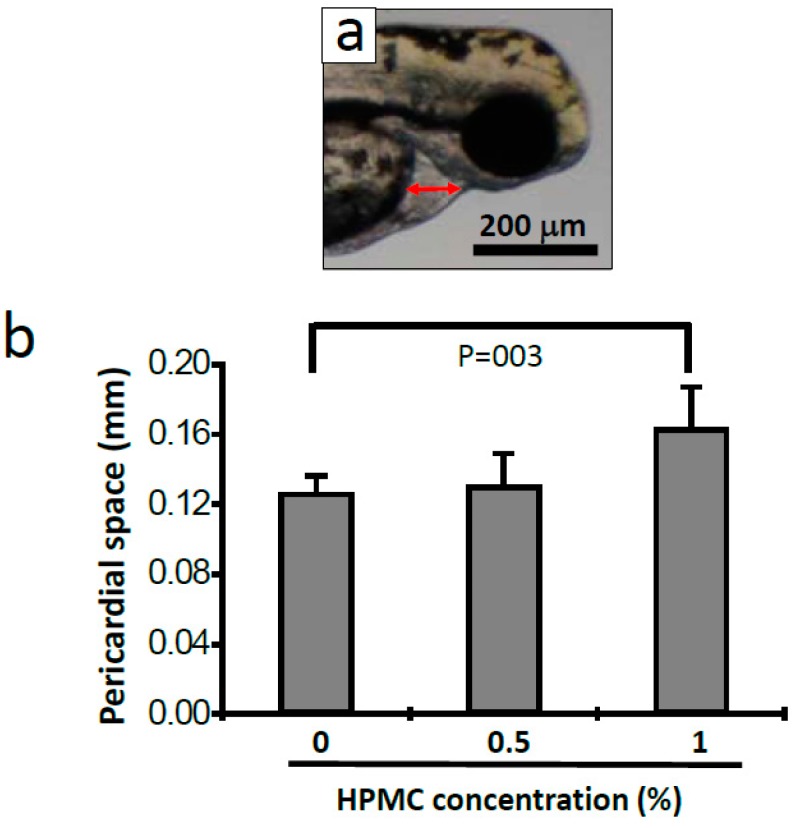
Pericardial lengths of zebrafish embryos at 48 hpf after HPMC treatment. (**a**) The red arrow indicates the length of the measured pericardial length; (**b**) Statistics of pericardial length after 0% (control), 0.5%, or 1% HPMC treatment. A significant difference between the 0% and 1% HPMC groups was determined (*p* = 0.03) through a Student’s *t* test. *n* = 7 in each treatment.

At 48 hpf after incubation in HPMC, the hatching rate, which is one of the phenotype markers of the FET test, decreased following the increase of the concentrations of HPMC (the numbers of non-hatching embryos were as follows: control = 1, 0.5% HPMC = 3, 1% HPMC = 12, *n* = 20 for each treatment). In addition, another phenotype marker, pericardial edema, was significantly increased in the 1% HPMC-treated embryos (Figure 7 and Figure 8, the pericardial length of the control = 0.13 ± 0.01 mm, 1% HPMC = 0.16 ± 0.02 mm, *p* = 0.003, *n* = 7 for each treatment). This suggested that the circulatory systems in the 48-hpf embryos were affected [34]. However, the 0.5% HPMC-treated embryos did not show any defects on their pericardial edemas. Aside from the pericardial edemas, we also simultaneously checked the heartbeat. However, no difference in heartbeat was observed after HPMC treatment. When we observed the HPMC-treated embryos at 72 hpf, we determined that most of the embryos had already hatched, and we observed no differences in heartbeat, tissue necrosis, or body length between the control and HPMC treatment groups.

In summary, concentrations of HPMC as high as 0.5% in the treatment exert no obvious effect on zebrafish embryo development. If the concentration is 1%, then not only does the shape of the chorion change, but also the hatching is delayed, the pericardial space in the embryo increases, and the circulatory system may exhibit malformation. Studies have indicated that some substances with molecular weight ≥3 kDa can delay the hatching rate [48], a finding that is similar to the findings of the present study. However, in this study, HPMC incubation did not cause somite malformation or mortality. The concentrations of HPMC that were used in the present study were far higher than those that can be detected in the environment. When used at the low concentrations that are typical of environmental levels, HPMC causes no mortality or very low mortality. In the present study, HPMC did not reach the 50% lethal concentration. This indicates that HPMC is an excellent biopolymer base for use in environmentally safe lubricants. In fact, HPMC is used as a component of artificial tears [49,50] and it has been tested in other toxicity assays [51,52,53,54].

## 3. Materials and Methods

### 3.1. Film Preparation

The HPMC film-making process involved heating 10–50 mL of water and 100 mL of alcohol to 60 °C, gently pouring them onto 5 g of HPMC (Pharmacoat 606, Shin-Etsu, Tokyo, Japan) and stirring the mixture until the latter completely dissolved. A micropipette was used to administer 150 μL of the solution slowly unto a silicon substrate that had been cleaned using alcohol, acetone, and isopropanol. The HPMC coated silicon substrate was then left to stand for 1 h in an environment with a temperature of 25 ± 2 °C and a relative humidity (RH) of 60% ± 5%.

### 3.2. Raman Spectroscopy and Eds Analysis

Film properties such as thickness and surface characteristics were analyzed using a SEM (JEOL, JSM-6700F, Peabody, MA, USA) and EDS, whereas thin film properties were assessed using the Raman spectrum (*i.e.*, Renishaw system 2000 micro-Raman spectrometer, Renishaw, New Mills, UK). Regarding the uniformity analysis, it involved randomly selecting nine points on the film and identifying the characteristic peak intensity of each point using the Raman spectrum; the peak value intensities were then recorded for a film uniformity comparison. A 3D scanner (Keyence, VK9710, Osaka, Japan) was applied for the thickness measurement.

### 3.3. Tribology Test

This study investigated the lubricating properties and antiwear behavior of 3.5% HPMC by using a ball-on-disk tribometer (Fu Li Fong precision Machine, Kaohsiung, Taiwan). For testing, a ball was fixed on a stationary holder, and the bottom disk was rotated at a specified speed. Tribology tests were performed in ambient air at ambient temperatures with a sliding speed variation of 0.01–0.10 m/s and loading variation of 2–8 N. All friction and wear tests were conducted at a rotation radius of 2 mm. The steel balls were composed of AISI 52100 with a hardness of 61HRC. The tested HPMC coating was prepared on a silicon substrate and mounted onto the bottom disk of the tribometer. The resistance to the motion of the disk (*i.e.*, the friction force) was recorded by connecting a load cell to the rotating disk. The friction coefficient was measured for further analysis of the lubrication and antiwear properties. The friction experiments were repeated to ensure the reproducibility of the results.

### 3.4. Zebrafish Embryo Toxicity Test

Zebrafish strain and husbandry: Wild-type zebrafish (*Danio rerio*, AB) were used and maintained under standard conditions, as described in Westerfield’s guidelines for the experimental use of zebrafish [55]. The present study was performed according to the animal use protocol of National Cheng Kung University and inspected by the Institutional Animal Care and Use Committee of National Cheng Kung University. All possible steps were taken to minimize animal discomfort. HPMC treatment and photography: Fertilized zebrafish embryos were incubated in different concentrations of HPMC immediately upon embryogenesis and until 72 hpf. Fish water was prepared by 0.3X Danieau buffer (cold spring harbor protocols [47]) and 1X penicillin/streptomycin (Gibco). The HPMC solutions were prepared by adding 0 g, 0.5 g, 1 g HPMC powder into the as-prepared fish water for different concentrations. The HPMC medium samples for the incubation were changed every day. The embryos were photographed at different developmental stages. The incubation temperature was maintained at 28 °C during the entire experiment. The HPMC-treated zebrafish embryos were anesthetized using 0.16 mg/mL Tricaine (Ethyl 3-aminobenzoate methanesulfonate salt, A5040, Sigma, St. Louis, MO, USA) during photography and were then released into the incubation medium. The photographs were taken using a Leica Z16 APO macroscope (Wetzlar, Germany) with a Canon DS126431 camera (Tokyo, Japan) and were analyzed using Image J software (1.48v, National Institute of Health, Bethesda, MD, USA).

## 4. Conclusions

(1) Dry films made from biopolymers feature an adjustable thickness and favorable uniformity.
(2) Compared with bare silicon frictions, silicon substrates coated with HPMC dry films demonstrate a superior lubrication result.
(3) The forming of transfer layers is the primary reason that HPMC demonstrates superior lubrication properties.
(4) HPMC is a biologically and environmentally friendly biopolymer material as well as a suitable and sustainable candidate for making green lubricants.

## Abbreviations

The following abbreviations are used in this manuscript:

NCKU	National Cheng Kung University
HPMC	Hydroxypropyl methylcellulose
SEM	Scanning electron microscope
EDS	Energy dispersive spectroscopy
COFs	Coefficient of frictions
FET	Fish Embryo Acute Toxicity
Hbf	Hours post fertilization
